# Aqueous Extract of *Salvia miltiorrhiza* Bunge-*Radix Puerariae* Herb Pair Attenuates Osteoporosis in Ovariectomized Rats Through Suppressing Osteoclast Differentiation

**DOI:** 10.3389/fphar.2020.581049

**Published:** 2021-01-21

**Authors:** Huan Qin, Wenwen Zhao, Yang Jiao, Haoyi Zheng, Hao Zhang, Jingyu Jin, Qiu Li, Xiuping Chen, Xia Gao, Yantao Han

**Affiliations:** ^1^ School of Basic Medical Sciences, Qingdao University, Qingdao, China; ^2^ State Key Laboratory of Quality Research in Chinese Medicine, Institute of Chinese Medical Sciences, University of Macau, Macao, China; ^3^ Department of Biomedical Engineering City University of Hong Kong, Hong Kong SAR, China; ^4^ Department of Pharmacology, School of Pharmacy, Qingdao University, Qingdao, China; ^5^ College of Chemistry and Pharmaceutical Sciences, Qingdao Agricultural University, Qingdao, China; ^6^ Qingdao Central Hospital, The Second Affiliated Hospital of Qingdao University, Qingdao, China

**Keywords:** osteoporosis, Salvia miltiorrhiza bunge-radix puerariae, autophagy, oxidative stress, osteoclast differentiation

## Abstract

Traditional herb pair *Salvia miltiorrhiza* Bunge-*Radix Puerariae* (DG) owns various biological activities including anti-inflammatory and anti-oxidative stress. Oxidative stress is one high-risk factor for osteoporosis, then effect of DG on osteoporosis and underlying mechanisms was explored both *in vivo* and *in vitro*. Firstly, the predication from network pharmacology hinted that DG has the potential for ameliorating osteoporosis. Consistent with predication, DG significantly restored bone loss and deficiency of type II collagen, decreased TRAP and Cathepsin K positive areas in femur. Meanwhile it improved important characteristics of microarchitectural deterioration of tissue, reduced the numbers of NFATc1-positive osteoclast in the vertebra as well as decreased the serum osteoclast-specific cytokine RANKL and OPG release in OVX rats exhibiting its protective effect against osteoporosis. *In vitro*, DG noticeably decreased osteoclastic-special marker protein expressions of RANK, c-Fos and NFATc1. Furthermore, autophagy pathway p62/LC3B, ROS production and NF-κB were all activated by RANKL stimulation and blocked by DG pretreatment. Moreover, autophagy inhibitors, ROS scavenger, Ca^2+^ chelator and NF-κB inhibitor remarkably suppressed c-Fos and NFATc1 expressions. Taken together, DG may ameliorate osteoporosis by regulating osteoclast differentiation mediated by autophagy and oxidative stress. This study provided a mechanistic basis for DG treating osteoporosis and offered a safe dose for DG in preventing and improving bone diseases.

**GRAPHICAL ABSTRACT:**
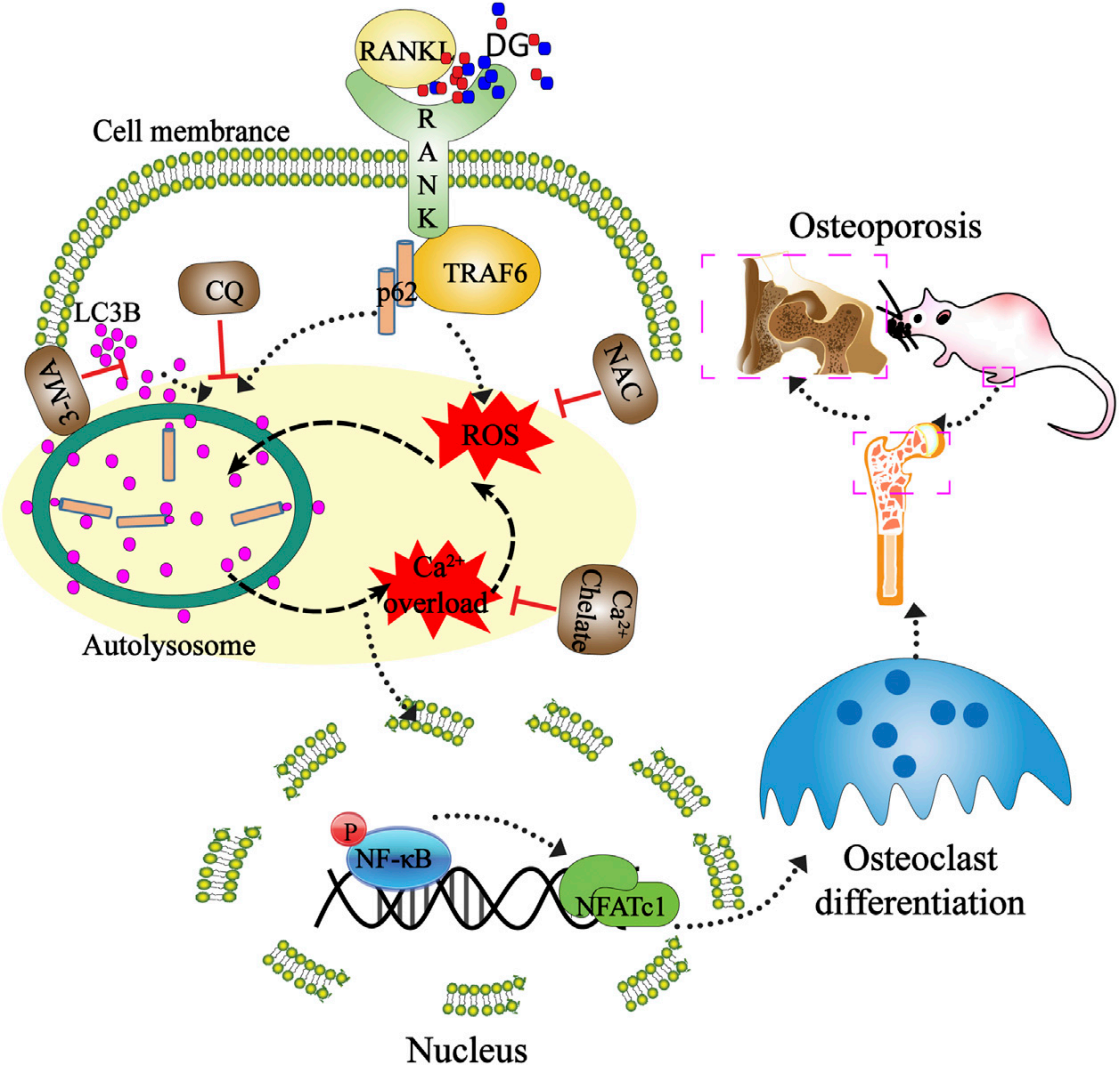
Mechanisms of DG ameliorating osteoporosis. DG improved osteoporosis by regulating osteoclast differentiation mediated by autophagy and oxidative stress. This study provided a mechanistic basis for the clinical application of DG in osteoporosis treatment.

## Introduction

Osteoporosis is a progressive skeletal disorder characterized by increased bone resorption and reduced bone mineral density (Mirza and Canalis, 2015). Nowadays excessive bone resorption due to enhanced osteoclast formation is accepted the most important cellular cause of osteolytic disease (Appelman-Dijkstra and Papapoulos, 2015) and osteoclast is considered as an important target for treating osteoporosis (Cackowski et al., 2010).

Macrophage colony-stimulating factor (M-CSF) and the receptor activator of nuclear factor kappa B ligand (RANKL) are two indispensable hematopoietic factors for osteoclast proliferation and mutation (Roux and Briot, 2018). Among these, RANKL binding to its receptor activates a series of downstream signal pathways including AKT-PLCγ (Ma et al., 2018), MAPKs(Li et al., 2018; Du et al., 2020) and NF-κB (Thummuri et al., 2015; Tu et al., 2020).

Accumulated studies showed that autophagy and oxidative stress are involved in the process of osteoporosis (Schröder, 2015; Shen et al., 2018; Wang et al., 2019). Autophagy is activated in response to several pro-inflammatory cytokines such as IL-1β and TNF-α accompanied by a robust increase in osteoclast differentiation and bone resorption (Wehmeyer et al., 2016; Jiang et al., 2019). Autophagy-related proteins including atg5, atg7, atg4β, and LC3B are identified involved in the formation of ruffled border and facilitation of osteoclast polarization, ultimately results in bone resorption (Hu et al., 2020). Besides, ROS is a critical intracellular signal mediator which induces osteoblast apoptosis through multiple cellular signaling pathways and identifies the osteogenesis deterioration (Atashi et al., 2015).

Nowadays, clinical drugs for treating osteoporosis cause serious adverse effects specially kidney injury (Liu et al., 2018). Besides, they also have ineffective prognosis for long-term treatments (Roux and Briot, 2018). Considering the characteristic of chronic diseases, there is an urge for seeking potentially compounds with low toxicity for long-term treatment of osteoporosis.

Recently, traditional herbs are attracting more and more attention owing to their low toxicity, few side effects as well as high safety for long-term treatment (Adler et al., 2016; Kelly et al., 2019). *Salvia miltiorrhiza* Bunge, one common traditional Chinese medicine, is widely used to treat dementia and cardiovascular diseases (Chen et al., 2019). *Pueraria montana* var. lobata (Willd.) Maesen and S.M.Almeida ex Sanjappa and Predeep (*Radix Puerariae*), another important medicine, has been reported to improve liver function (Feng et al., 2019), enhance alcohol detoxification processes, regulate cardiac functions (Tan et al., 2017), as well as aid weight loss (Adler et al., 2016). Our previous study showed that herb pair *Salvia miltiorrhiza* Bunge-*Radix Puerariae* (DG) apparently improved vascular injury in diabetic mouse model by reducing oxidative stress (Zhao et al., 2019). In view of the fact that oxidative stress takes part in regulating activity and function of osteoblasts and osteoclasts cells (Forte et al., 2017), the effects of DG on osteoporosis and related mechanisms were explored both *in vivo* and *in vitro*.

## Materials and Methods

### Chemical Compounds and Reagents

*Salvia miltiorrhiza* Bunge and *Radix Puerariae* were purchased from Changda Prepared Chinese Medicinal Herbs Co. Ltd (Anguo, China). Healthy SD rats, SPF clean grade, female, 10 weeks old, weight 200–250 g, were purchased from Chinese Academy of Sciences; The ELISA kits for detecting blood urea nitrogen (BUN), creatinine, ALP, OPG and RANKL were purchased from Wuhan Huamei Biological co., LTD (Wuhan, China); DCFH2-DA, Fluo-3/AM, BAPTA-AM and Hoechst 33,258 were obtained from Molecular Probes (Eugene, OR); Chemicals used for DPI, TTRA, AA, ALL, NAC, NDGA, Rot were purchased from Sigma-Aldrich (St. Louis, MO). H&E and Masson dying kits were purchased from Nanjing Jiancheng Biological Engineering Research Institute (Nanjing, China); 3-methyladenine (3-MA), and monodansyl cadaverine (MDC) were obtained from MedChemExpress; TRAP staining kit was obtained from BIO-SCIENCE COMPANY LIMITED (Shanghai, China); Antibodies GAPDH, LC3B, Beclin-1, p62, p-p65 and NF-κBp65 were purchased from Sigma Company; Antibodies NFATc1 and c-Fos were purchased from Chengdu Zen Bio (Chengdu, China); Secondary antibodies were purchased from American CST Company; Hoechst 33,342 was purchased from Thermo Fisher Scientific; JYB1-1 calcium removal solution and protein extraction kit were purchased from Solarbio Company (Beijing, China); RANKL was purchased from Sino Biological Company (Beijing, China).

### Preparation of DG

The dried *Salvia miltiorrhiza* Bunge and *Radix Puerariae* were crushed into powder and 500 g powder (1:1 w/w; 250 g each) was put into 6,000 ml water at 25°C for 30 min and then extracted under 100°C for 45 min. The extraction procedure was repeated. After freeze drying, the extract was produced into powder and stored at 4°C.

### Analysis of DG Constitutes

The exact constitutes of DG was detected with HPLC. Detail experimental conditions and information are prepared as described previously (Zhao et al., 2019) (Supplementary Figure S2).

### Animal Experiments and Ethical Statement

Forty specific pathogen-free SD rats were approved by the Animal Experimentation Ethics Committee of Chinese Medicine. All animal procedures followed the NIH guide for the Care and Use of Laboratory Animals (NIH Publications No. 80-23, revised 1978). One week after the rats arrived, 10 female rats in the same physiological condition underwent bilateral laparotomy (Sham), and the other 30 underwent bilateral ovariectomy (OVX). OVX female rats were further randomly assigned into three groups (*n* = 10/group): vehicle group, DG (50 mg/kg/d; *n* = 10), DG (200 mg/kg/d; *n* = 10) (with orally administrated DG treatment for 8 weeks). After finishing the trials, blood was collected from aorta abdominalis with 15 ml injector and centrifuged at 3,000 rpm for 10 min. Then supernatant was stored at 80°C for biochemical analyses. The left femur samples were removed by gauze and fixed in 4% paraformaldehyde overnight at 4°C, then stored in 70% ethanol for micro-CT scanning. The right femur samples and lumbar vertebras were obtained and fixed in 4% paraformaldehyde for histological detection. Then each group’s sections were taken for staining by H&E, Masson and Immunohistochemistry and the processes strictly followed the instructions of the kits.

### Histomorphometric Analysis by Micro-CT

After mice were sacrificed, their left femurs were scanned and trabecular morphometric analysis were performed on a micro-CT imaging system (Skyscan 1,176, Belgium). The trabecular region of interest was selected and analyzed quantifying BMD, BMC and morphometric calculations. Related parameters including bone volumetric fraction (BV/TV, %), trabecular thickness (Tb.Th, mm), trabecular separation (Tb.Sp, mm), and trabecular number (Tb.N, mm^−1^) were measured.

### ELISA Assay

The secretion levels of urea nitrogen (BUN), creatinine, osteoprotegerin (OPG), alkaline phosphatase (ALP) and RANKL in serum were determined using ELISA kits following the instructions of manufacture. Each specimen was repeated 3 times and averaged.

### Cell Culture

RAW 264.7 cell line was obtained from American Type Culture Collection (ATCC, Rockville, United States). Cells were cultured in DMEM supplemented with 10% FBS, penicillin (100 μg/ml) and streptomycin (100 μg/ml) in a humidified incubator with 5% CO_2_ at 37°C.

### TRAP Staining

In the experiment, on the 5^th^ day, cells were fixed with 4% paraformaldehyde in PBS for 20 min at room temperature and then washed with PBS. After natural drying, TRAP fixation solution was fixed for 3 min at 4°C and slightly dried after washing. TRAP incubation solution was added and placed in an incubator avoiding light at 37°C for 60 min. Then plants were dyed with hematoxylin solution for 3–5 min, later washed and dried for microscopic examination (Olympus Corporation of the Americas, Waltham, MA, ix81). Finally, TRAP-positive multinucleated cells (purple) with at least five nuclei were counted as osteoclasts.

### Network Pharmacology Predicts the Mechanism of DG in Treating Osteoporosis

Inputting DG into the symmap (http://www.symmap.org/) database, we got 615 symptoms and 60 targets. The symptoms and targets were screened by this network. In this network, we selected the symptoms of osteoporosis and targets involved in NF-κB inflammatory.

### MDC Staining

MDC was used as a specific autophagolysosome marker to analyze the autophagic process. RAW264.7 cells were cultured on dishes at a density of 1×10^4^ cells/mL overnight and then stimulated with RANKL (50 ng/ml) for 1–7 days, cells were incubated with 50 mM MDC at 37°C for 15 min and washed with 1 × PBS three times with 5 min interval. Finally, the cells were observed under the confocal laser scanning microscopy.

### Western Blot Analysis

Treated RAW264.7 cells were lysed with RIPA buffer supplemented with phosphatase inhibitors. Cell lysates were separated using 8–12% SDS-PAGE and then transferred onto PVDF membranes. After membranes were blocked with 5% non-fat milk in TBST (20 mM Tris-HCl, 500 mM NaCl, and 0.1% Tween 20) at room temperature for 1 h, membranes were incubated with targeted primary antibodies and corresponding secondary antibodies. Lastly, chemiluminescence signals were detected with a ChemiDoc™ Imager.

### Statistical Analysis

The data were expressed as the means ± SD. Statistical analyses were performed by one-way ANOVA followed by Tukey post-hoc test. Values of *p* < 0.05 were considered statistically significant.

## Results

### Effect of DG on OVX-Induced Osteoporosis in Rats

**FIGURE 1:**
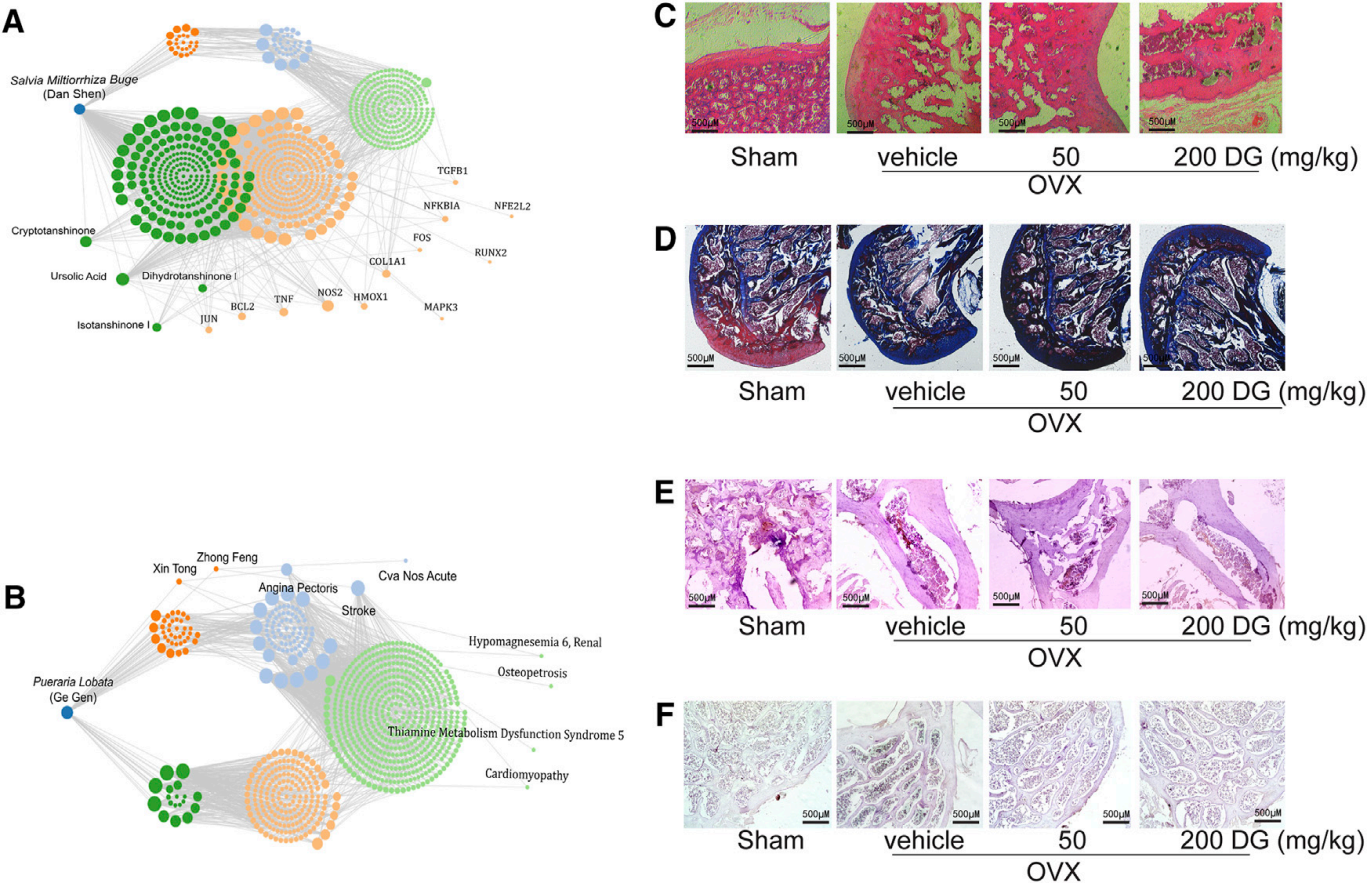
DG restored the bone loss and the deficiency of type II collagen in femur from osteoporosis rats **(A and B)**. Data from network pharmacology analysis of *Salvia miltiorrhiza Bunge-Radix Puerariae herb* Rats were treated as described in MATERIALS AND METHODS section, **(C)** morphological changes of bone loss in femur were detected by HE staining (magnification × 40), **(D)** collagen content in femur was detected by Masson staining (magnification × 40), **(E)** TRAP-positive area was detected by TRAP staining (magnification × 40) and **(F)** Cathepsin K-positive area was detected by Immunohistochemistry (magnification × 40). DG, aqueous extract of *Salvia miltiorrhiza Bunge-Radix Puerariae* herb pair.

**FIGURE 2:**
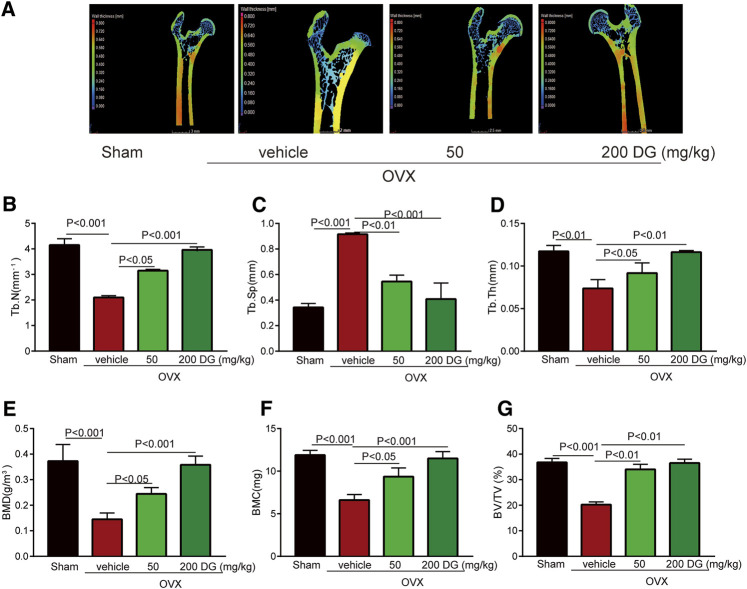
DG improved characteristics of microarchitectural deterioration of tissue in osteoporosis rats **(A)**. After rats were treated, left femur samples were isolated for micro-CT scanning. **(E)** Characteristics of bone microarchitecture in femur including bone mineral density (BMD, g/m^3^), **(F)** bone mineral content (BMC, mg), **(G)** bone volume fraction (BV/TV, %), (C) trabecular separation (Tb.Sp, mm), **(D)** trabecular thickness (Tb.Th, mm) and **(B)** trabecular number (Tb.N, mm^−1^) were detected. DG, aqueous extract of *Salvia miltiorrhiza Bunge-Radix Puerariae* herb pair.

To identify the prediction from network pharmacologic analysis that DG has potential on improving osteoporosis (Figures 1A,B), effects of DG on osteoporosis were evaluated *in vivo*. Firstly, the rat model of OVX-induced osteoporosis was successfully established. Morphologically, bone loss (Figure 1C), deficiency of type II collagen (Figure 1D), TRAP-positive area (Figure 1E) and Cathepsin K-positive area (Figure 1F) were significantly observed in the femur of OVX group while these phenomena were remarkably restored with DG administration (Figures 1C–F). Secondly, data analysis from micro-CT revealed a significant decrease in bone mineral density (BMD), bone mineral content (BMC), bone volume fraction (BV/TV), trabecular separation (Tb.Sp), trabecular thickness (Tb.Th) and trabecular number (Tb.N) as well as an increase in trabecular separation (Tb.Sp), which were important characteristics of microarchitectural deterioration of tissue. As expected, these phenomena are remarkably reversed by DG administration (Figure 2). In all, current data indicated the potential of DG on ameliorating osteoporosis.

### Mechanisms of DG on Protecting Against OVX-Induced Osteoporosis

**FIGURE 3:**
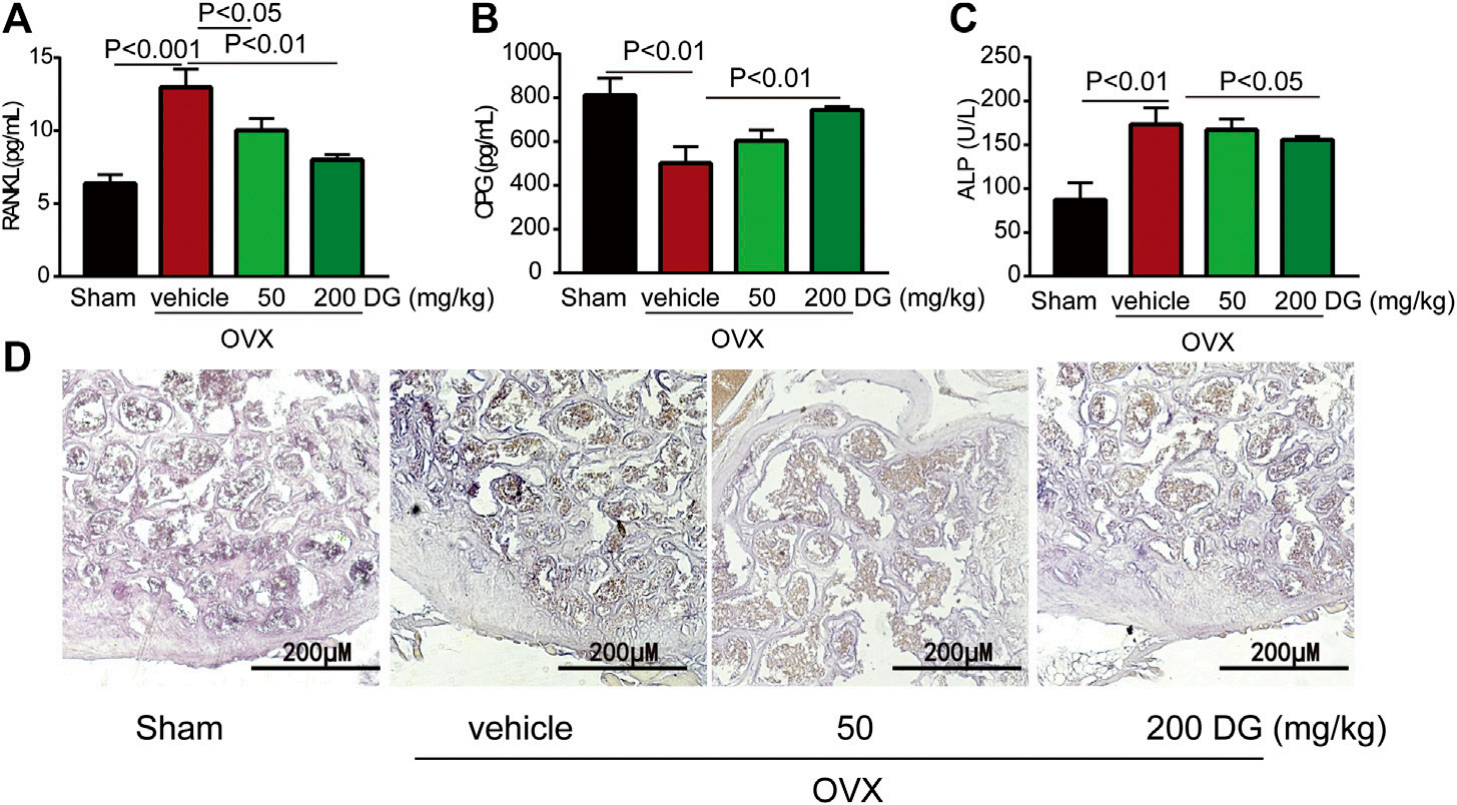
DG suppressed osteoclast-mediated bone loss. After rats were treated, serum was collected. **(A)** Serum RANKL **(B)** OPG and **(C)** ALP were detected by ELISA kits. Besides, **(D)** right femur samples were isolated for NFATc1 staining (magnification × 200). DG, aqueous extract of *Salvia miltiorrhiza Bunge-Radix Puerariae* herb pair.

**FIGURE 4:**
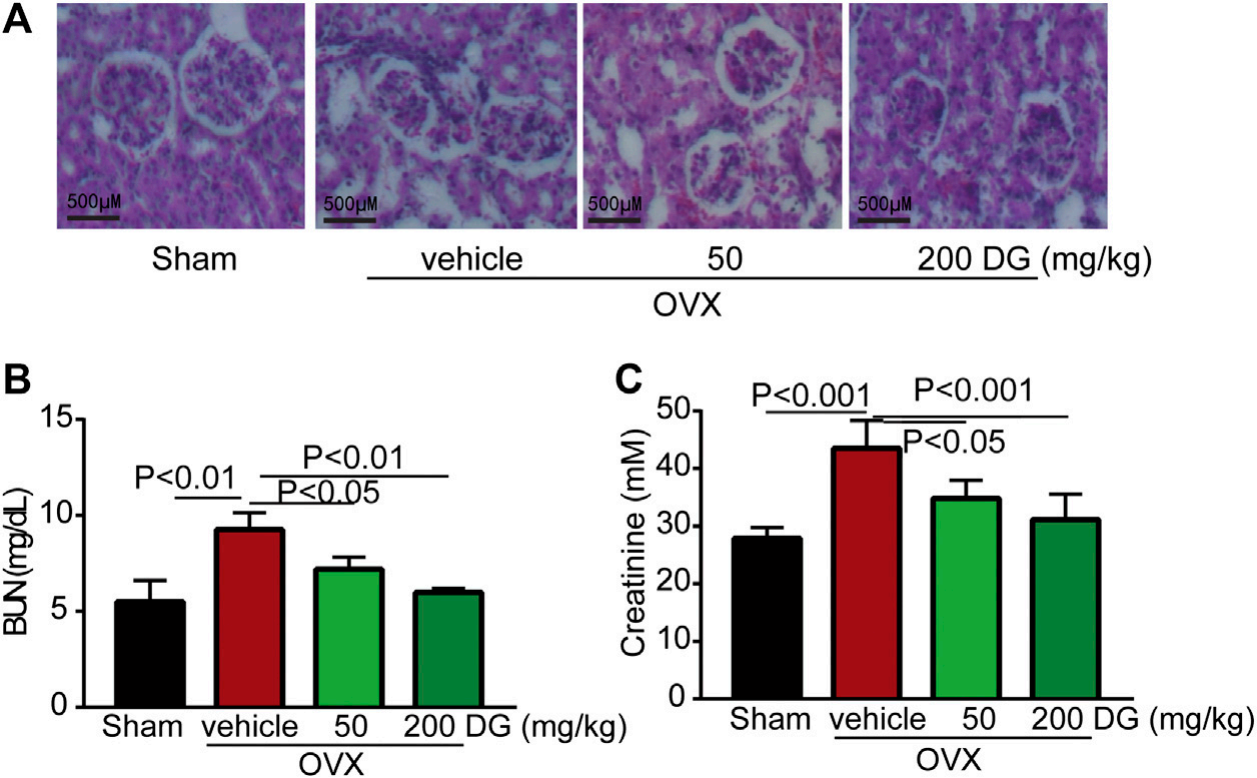
DG ameliorated kidney injury in rats with osteoporosis. After rats were treated, kidneys were completely isolated. **(A)** Morphological changes of glomerulus were assessed with HE staining, **(B)** kidney function indexes as BUN and **(C)** creatinine were also detected by ELISA kits. DG, aqueous extract of *Salvia miltiorrhiza Bunge-Radix Puerariae* herb pair.

The maintenance of bone homeostasis during bone remodeling and modeling was highly dependent on the stable modulation of osteoclast and osteoblast. In present study, data from serum ELISA tests revealed that DG apparently decreased the release of osteoclastogenesis markers RANKL and OPG instead of increasing osteogenesis marker ALP (Figures 3A–C) indicating that DG treated osteoporosis mainly by inhibiting osteoclastogenesis and this suppose was further identified by IHC staining of NFATc1 (Figure 3D). Currently, kidney injury is considered as one serious adverse effect of clinical drugs for treating osteoporosis. As shown in Figure 4, DG administration significantly ameliorated chronic kidney injury by improving glomerular atrophy, decreasing BUN and creatinine release levels in rats from OVX group demonstrating its advantage in the clinic.

### Effect of DG on RANKL-Induced Osteoclast Differentiation in RAW264.7 Cell Line

**FIGURE 5:**
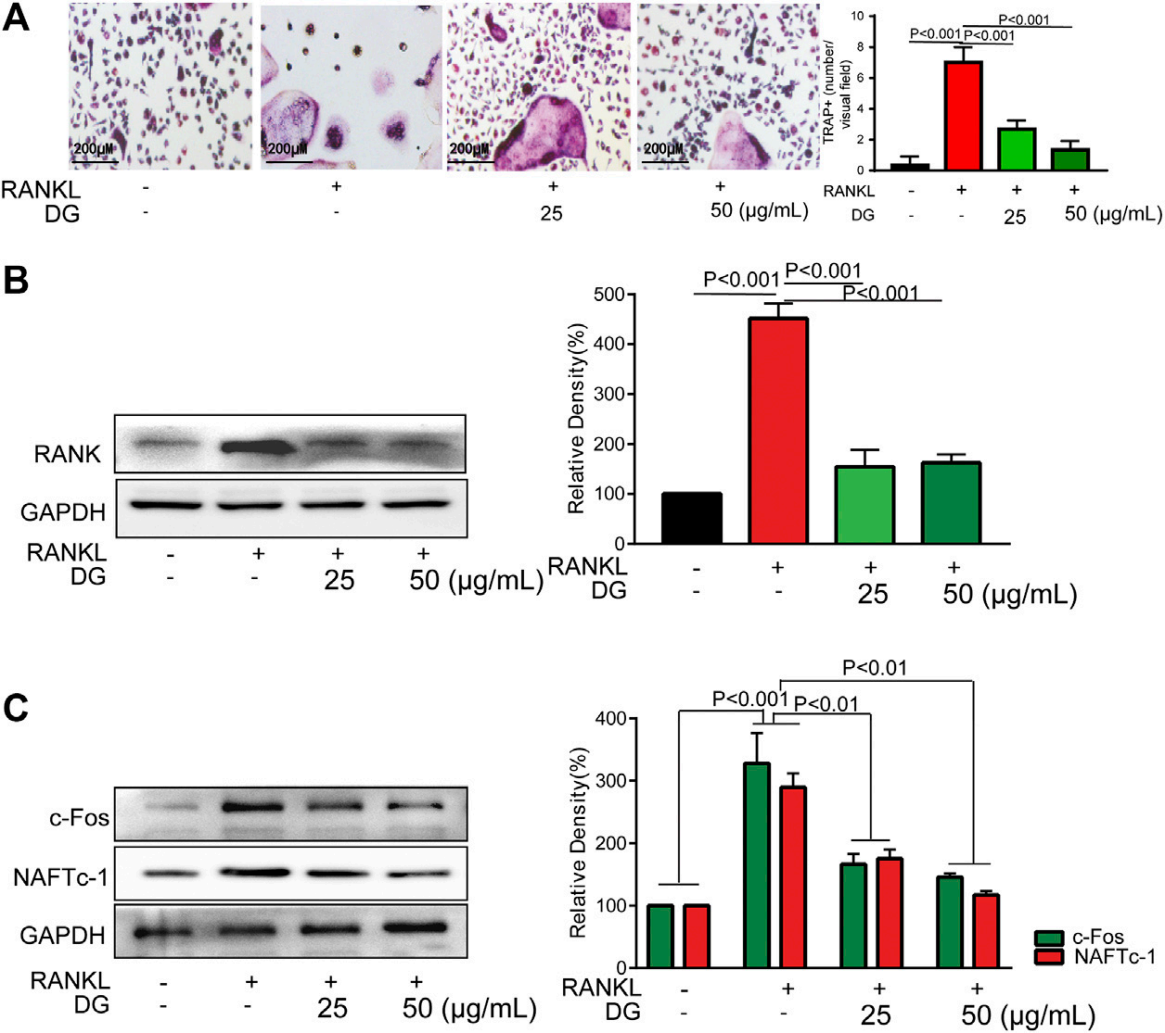
DG inhibited osteoclast differentiation in RAW264.7 cells. RAW264.7 cells were cultured for 5 days with RANKL (50 ng/ml) in the presence of DG (25, 50 μg/ml) for 1 h, **(A)** cells were stained for TRAP (magnification × 200) and **(B)** related proteins including RANK, **(C)** osteoclastic-special proteins c-Fos and NFATc1 were detected by western blotting. Representative images were acquired under a light microscope. DG, aqueous extract of *Salvia miltiorrhiza Bunge-Radix Puerariae* herb pair.

To further investigate the mechanisms of DG on preventing against osteoporosis, *in vitro* studies have been done. Firstly, to avoid the damage of DG with high concentration on cells, we examined the cytotoxicity of DG in RAW264.7 cells using the MTT assay (Supplementary Figure S1). The data showed that DG had no cytotoxic effect at present concentration 50 μg/ml. Then, a standard osteoclast differentiation model was established in RAW264.7 cells with stimulation RANKL. Formation of TRAP-positive multinucleated osteoclasts were obviously observed in RANKL-treated group. Meanwhile DG pretreatment significantly suppressed above changes (Figure 5A). Furthermore, DG decreased the expression of osteoclast-specific proteins RANK, NFATc1 and c-Fos (Figures 5B,C).

### Participation of Autophagy, Oxidative Stress and NF-κB in the Process of Osteoclast Differentiation

**FIGURE 6:**
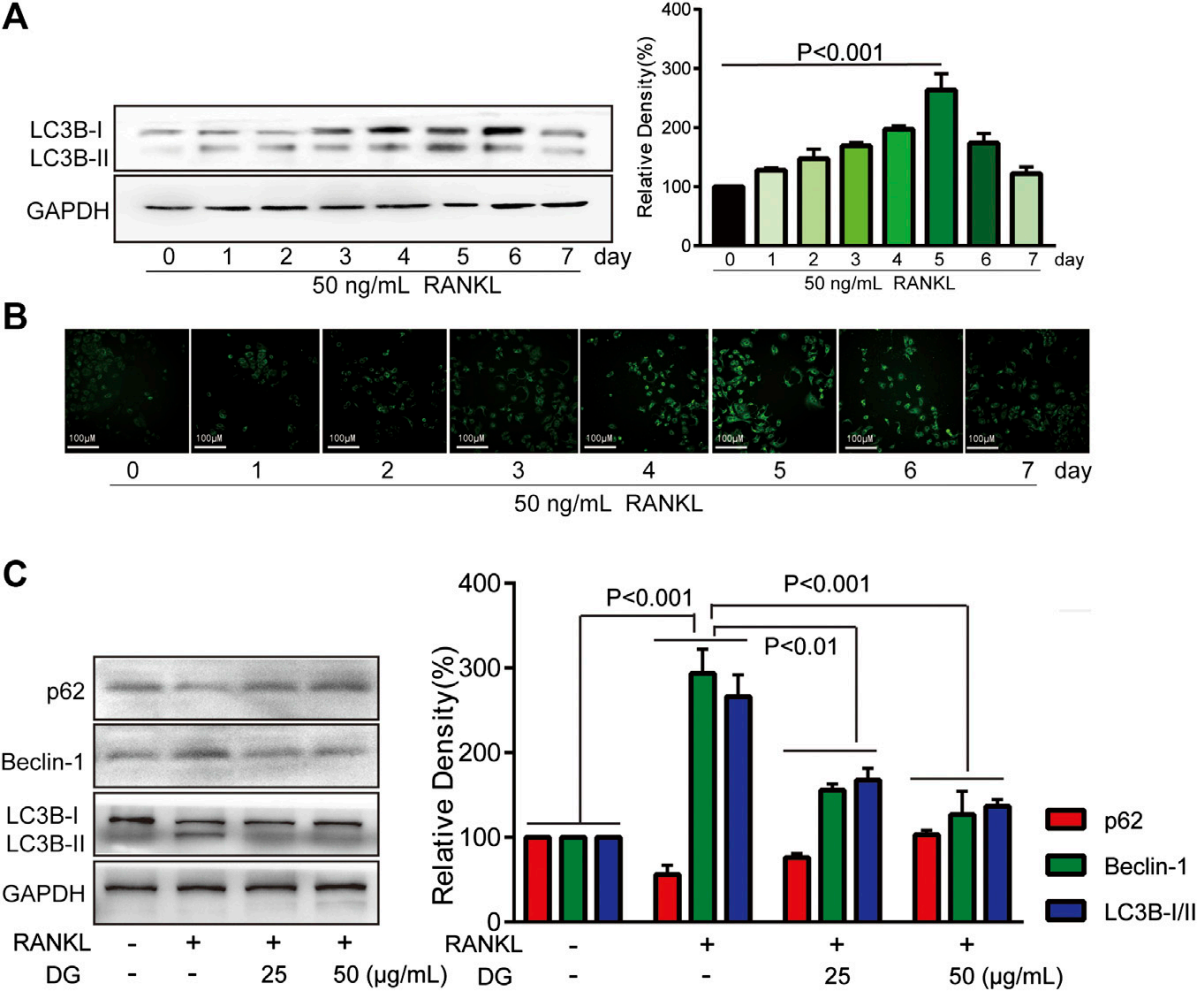
DG suppressed RANKL-induced autophagy signaling pathway. RAW264.7 cells were pretreated with RANKL (50 ng/ml) for 0, 1, 2, 3, 4, 5, 6, 7 days, **(A)** LC3BI/II expression was detected by western blotting and **(B)** autophagy vacuole was detected by MDC staining. **(C)** Then RAW264.7 cells were cultured for 5 days with RANKL (50 ng/ml) in the presence of DG (25, 50 μg/ml) for 1 h or not, autophagy-related proteins were detected by western blotting. DG, aqueous extract of *Salvia miltiorrhiza Bunge-Radix Puerariae* herb pair.

**FIGURE 7:**
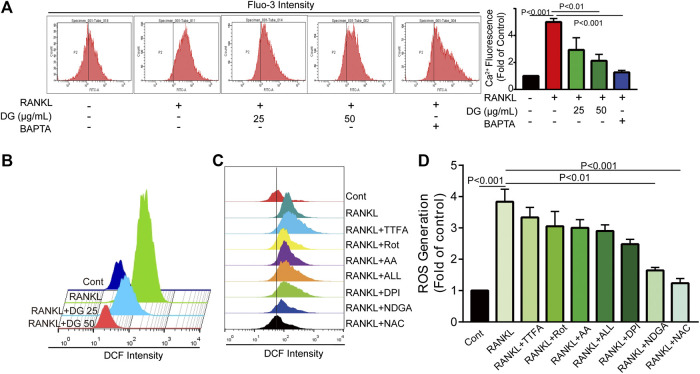
DG suppressed RANKL-induced ROS production and Ca^2+^ overload. The cells were pretreated with DG (50 μg/ml), BAPTA (10 μM), TTFA (10 μM), Rot (10 μM), AA (100 nM), ALL (5 μM), DPI (100 nM), NDGA (10 μM) and NAC (5 mM) separately for 1h and then stimulated with RANKL (50 ng/mL) for 5 days, **(A)** Ca^2+^ overload and **(B-D)** ROS production were determined by flow cytometry. AA, antimycin; ALL, allopurinol; BAPTA, BAPTA-AM; DG, aqueous extract of *Salvia miltiorrhiza Bunge-Radix Puerariae* herb pair; DPI, diphenyleneiodonium chloride; NAC, N-acetyl-L-cysteine; NDGA, nordihydroguaiaretic acid; Rot, rotenone; TTFA, 2-thenoyltrifluoroacetone.

**FIGURE 8:**
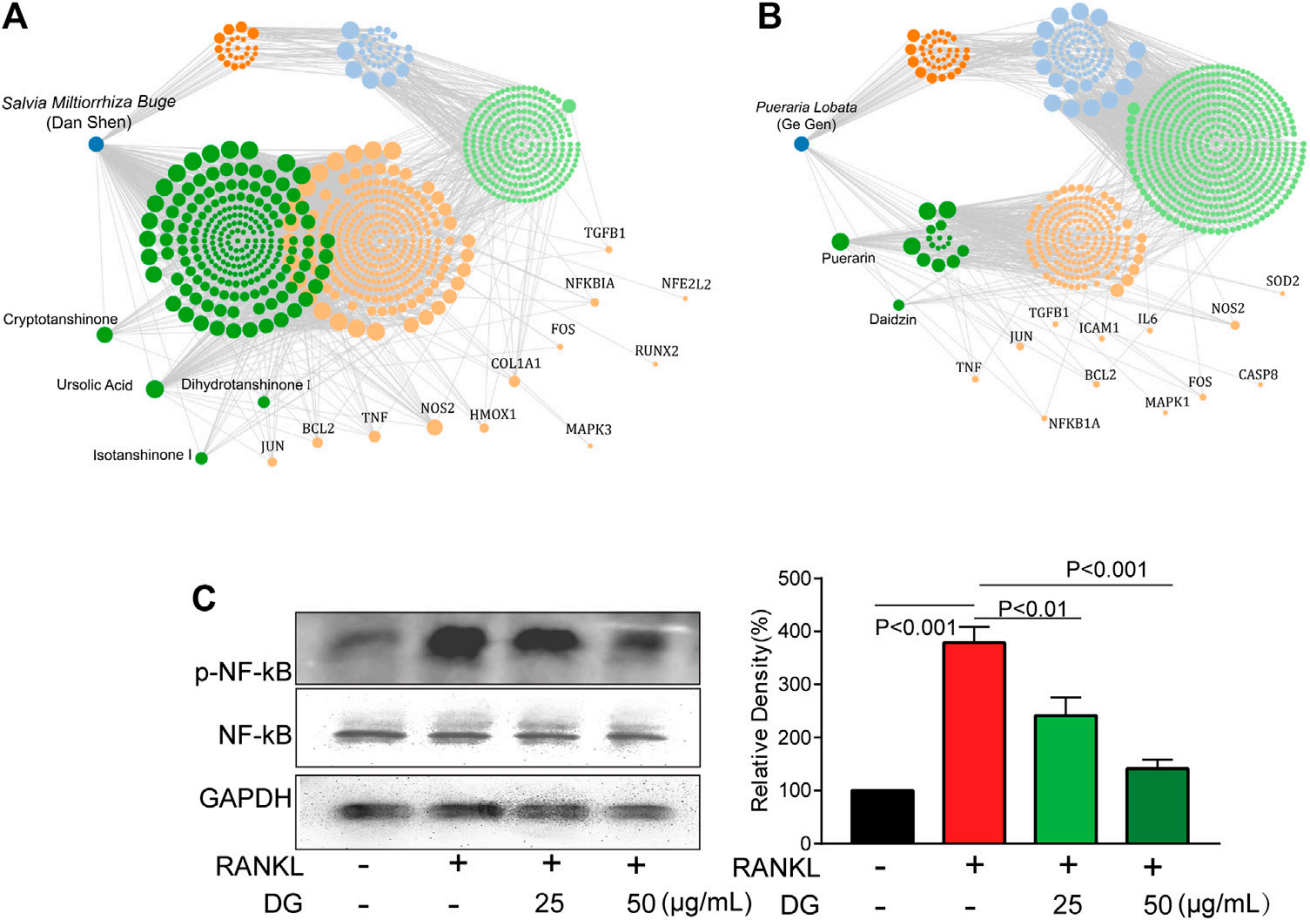
DG suppressed NF-κB pathway **(A and B)** Data from network pharmacology analysis of *Salvia miltiorrhiza Bunge-Radix Puerariae* herb. **(C)** RAW264.7 cells were pretreated with DG (25, 50 μg/ml) for 1h in response to RANKL (50 ng/ml) for 5 days, expressions of p-NF-κBp65 and NFκB were detected by western blotting. DG, aqueous extract of *Salvia miltiorrhiza Bunge-Radix Puerariae* herb pair.

**FIGURE 9:**
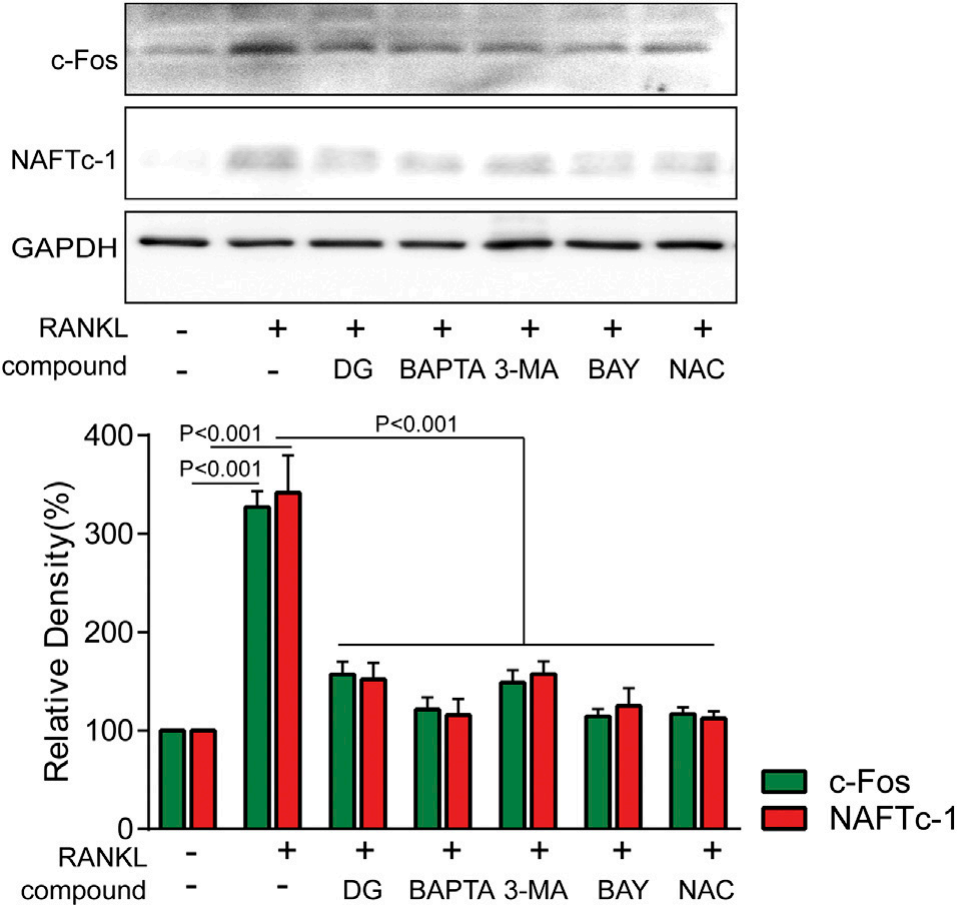
DG suppressed RANKL-induced osteoclast differentiation mediated by autophagy, oxidative stress and NF-κB RAW264.7 cells were pretreated with DG (50 μg/ml), BAPTA (10 μM), 3-MA (1 mM), BAY (10 μM) and NAC (5 mM) for 1 h in response to RANKL (50 ng/ml) for 5 days, osteoclastic-special proteins c-Fos and NFATc1 were detected by western blotting. BAPTA, BAPTA-AM; BAY , BAY11-7082; DG, aqueous extract of *Salvia miltiorrhiza Bunge-Radix Puerariae* herb pair; 3-MA, 3-methyladenine; NAC, N-acetyl-L-cysteine.

Firstly, in our study, LC3B I/II ratio was significantly up-regulated in a time-dependent manner with RANKL stimulation and LC3B-II got the highest expression on fifth day (Figure 6A). Meanwhile autophagic vesicles dyed by MDC staining was markedly observed on fifth day (Figure 6B). Furthermore, autophagy signaling pathway including increased Beclin-1, LC3B and decreased p62/SQSTM1 were also activated in response to RANKL stimulation. However, DG pretreatment obviously reversed these phenomena (Figure 6C). Then lipoxygenases-derived ROS production and Ca^2+^ overload were obviously induced by RANKL stimulation and reduced by DG pretreatment (Figure 7). Besides, data from network pharmacology predicts that *Salvia miltiorrhiza* Bunge and *Radix Puerariae* suppress NF-κB activity (Figures 8A,B). Consistent with the predictability by software, DG significantly inhibited RANKL-stimulated NF-κBp65 phosphorylation (Figure 8C). Lastly, DG and specific inhibitors for ROS, autophagy, and NF-κB all significantly decreased osteoclast-specific proteins NFATc1 and c-Fos expressions (Figure 9). Generally, our study showed that DG protected against OVX-induced osteoporosis in rats by suppressing osteoclast differentiation mediated by autophagy, oxidative stress and NF-κB (Supplementary Figure S1).

## Discussion

Considering serious adverse reactions caused by clinical osteoporosis drugs (Su et al., 2019; Chen et al., 2020; Hu et al., 2020), seeking potential drugs with hypotoxicity is one novel therapeutic strategy. In recent years, a large number of studies have proved the beneficial effect of Chinese herbal medicine in the treatment for osteoporosis. Polysaccharides isolated from lotus leaves exert anti-osteoporotic effects by inhibiting osteoclastogenesis (Hwang et al., 2020). Catalpol, the primary active principle component of Rehmanniae Radix, promotes the osteogenic differentiation of bone marrow mesenchymal stem cells via the Wnt/β-catenin pathway (Zhu et al., 2019). Polysaccharides from the roots of *Morinda* officinalis induce bone formation by up-regulating the expression of runt-related transcription factor 2, osterix, osteopontin, and osteocalcin (Yan et al., 2019). Resveratrol promotes osteogenesis via activating SIRT1/FoxO1 pathway in osteoporosis mice (Hwang et al., 2019). *Salvia miltiorrhiza* Bunge (Danshen) and *Radix Puerariae* (Gegen) are two valued traditional Chinese medicine for multiple therapeutic remedies. A classical herbal pair contains both Danshen and Gegen has a history of usage in China for treating a series of diseases including cardiovascular disease, diabetes and aging-induced diseases (Lim et al., 2013; Guo et al., 2014). In this study, protective effects and mechanisms of this herb pair on osteoporosis were explored.

Firstly, in *in vivo* study, bone destruction, microarchitectural deterioration and decreased bone density of femur bone were occurred in OVX group while DG significantly reversed these changes hinting DG’ potential for improving osteoporosis. Furthermore, DG balanced the release levels of serum osteoclast-specific marker RANKL and OPG as well as decreased NFATc1-positive osteoclasts in lumbar vertebra in OVX group. These data hinted that possible mechanisms for DG ameliorating osteoporosis was to suppress osteoclast activities. Chronic kidney injury is accepted one serious adverse effect for mostly clinical drugs in treating bone disorder diseases. In our present study, we deliberately detected kidney function of rats in different groups. Intriguingly, compared with OVX group, DG apparently improved kidney function exhibiting its advantage for being ideal clinical application.

### Clinically

Furthermore, in *in vitro* studies, RANKL-induced osteoclast formation accompanied by increased proteins expressions of osteoclastic-special marker c-Fos and NFATc1 were extensively suppressed with DG pretreatment. Then autophagy was occurred with RANKL stimulation evidenced by occurrence of autophagic vesicles and activation of autophagy related marker proteins while DG pretreatment significantly reversed these phenomena. Besides, DG evidently reduced RANK-induced ROS production. There are a variety of sources for ROS including NADPH oxidases, xanthine oxidases, arachidonic acid by lipoxygenases, and the mitochondrial respiratory chain (Mailloux et al., 2013; Aviello and Knaus, 2018; Mazat et al., 2020). In present study, NDAPH oxidase inhibitor diphenyleneiodonium chloride (DPI), xanthine oxidases inhibitor allopurinol (ALL), mitochondria respiratory electron-transport chain inhibitors antimycin (AA), rotenone (Rot) and 2-thenoyltrifluoroacetone (TTFA) had no significant scavenging effect response to RANK-induced ROS generation while lipoxygenases inhibitor nordihydroguaiaretic acid (NDGA) had similar scavenging capacity with DG hinting lipoxygenases was the main source of ROS generation induced by RANK. Besides, NF-κB is considered an activator of NFATc1 and c-Fos (Guo et al., 2019; Zhang et al., 2019). Data analysis from our network pharmacology showed that DG had a potential for inhibiting NF-κB. In practical experiments, DG did block RANKL-induced NF-κB/p65 activation. Finally, autophagy inhibitor, ROS scavenger, Ca^2+^ chelator and NF-κB inhibitor significantly suppressed osteoclast differentiation.

## Conclusion

Our *in vivo* research reveals DG’s protective effect against osteoporosis in OVX rats. *In vitro*, DG inhibits osteoclast differentiation. All autophagy, ROS and NF-κB are all involved in this process and regulated by DG. Present study offers scientific support for DG’s widely application for treating age-ralated diseases as health care products or therapeutic drugs.

## Data Availability

The raw data supporting the conclusions of this article will be made available by the authors, without undue reservation, to any qualified researcher.

## Abbreviations

AA, antimycin; ALL, allopurinol; ALP, alkaline phosphatase; BAPTA, BAPTA-AM; BAY, BAY11–7,082; BUN, blood urea nitrogen; CQ, chloroquine; DG, aqueous extract of Salvia miltiorrhiza Bunge-Radix Puerariae herb pair; DPI, diphenyleneiodonium chloride; M-CSF, macrophage colony stimulating factor; MDC, monodansylca daverine; NAC, N-acetyl-l-cysteine; NDGA, nordihydroguaiaretic acid; NFATc1, nuclear factor of activated T-cells, cytoplasmic 1; OPG, osteoprotegerin; OVX, ovariectomy; RANKL, receptor activator of nuclear factor kappa-B ligand; Rot, rotenone; TRAP, tartrate-resistant acid phosphatase; TRAF6, TNF receptor associated factor 6; TTFA, 2-thenoyltrifluoroacetone; 3-MA, 3-methyladenine
